# Evaluation of Impedance-Based Label-Free Technology as a Tool for Pharmacology and Toxicology Investigations

**DOI:** 10.3390/bios3010132

**Published:** 2013-03-15

**Authors:** Franck André Atienzar, Helga Gerets, Karen Tilmant, Gaëlle Toussaint, Stéphane Dhalluin

**Affiliations:** UCB Pharma SA, Non Clinical Development, Chemin du Foriest, 1420 Braine-l’Alleud, Belgium; E-Mails: helga.gerets@ucb.com (H.G.); karen.tilmant@ucb.com (K.T.); gaelle.toussaint@ucb.com (G.T.); stephane.dhalluin@ucb.com (S.D.)

**Keywords:** real-time cell analyzer (RTCA), impedance, drug discovery, investigative pharmacology, predictive toxicology, screening

## Abstract

The use of label-free technologies based on electrical impedance is becoming more and more popular in drug discovery. Indeed, such a methodology allows the continuous monitoring of diverse cellular processes, including proliferation, migration, cytotoxicity and receptor-mediated signaling. The objective of the present study was to further assess the usefulness of the real-time cell analyzer (RTCA) and, in particular, the xCELLigence platform, in the context of early drug development for pharmacology and toxicology investigations. In the present manuscript, four cellular models were exposed to 50 compounds to compare the cell index generated by RTCA and cell viability measured with a traditional viability assay. The data revealed an acceptable correlation (*ca*. 80%) for both cell lines (*i.e*., HepG2 and HepaRG), but a lack of correlation (*ca*. 55%) for the primary human and rat hepatocytes. In addition, specific RTCA profiles (signatures) were generated when HepG2 and HepaRG cells were exposed to calcium modulators, antimitotics, DNA damaging and nuclear receptor agents, with a percentage of prediction close to 80% for both cellular models. In a subsequent experiment, HepG2 cells were exposed to 81 proprietary UCB compounds known to be genotoxic or not. Based on the DNA damaging signatures, the RTCA technology allowed the detection of *ca*. 50% of the genotoxic compounds (n = 29) and nearly 100% of the non-genotoxic compounds (n = 52). Overall, despite some limitations, the xCELLigence platform is a powerful and reliable tool that can be used in drug discovery for toxicity and pharmacology studies.

## 1. Introduction

In drug development, *in vitro* approaches play an important role for the selection of efficacious and safe candidates before animal studies are launched [1,2]. At UCB, a battery of *in vitro* assays focusing on cytotoxicity [3], phospholipidosis [4] and micronuclei detection [5] were validated for screening purposes. Recently, several clinically promising cytotoxic and cytoprotective agents with potential applications in cancer, ischemic and neurodegenerative diseases have been identified by high-throughput screening (HTS), based on appropriate cell death assays [6]. Many *in vitro* assays are available to identify potential toxic liabilities, but the vast majority of the assays are invasive and measurements are performed at fixed time points (e.g., 24 h). Such an approach is not optimal because, for instance, apoptosis, which usually occurs within a few hours, is frequently followed by secondary necrosis events that may take place immediately or in a longer time frame. In addition, induced cell cycle arrest may be temporary, while in other cases the cells could be permanently blocked leading finally to cell death. Consequently, the use of label-free technologies (e.g., the xCELLigence platform based on impedance as readout), which allow continuous measurements, are receiving more and more attention [7,8]. For instance, recently, Kustermann *et al*. [8] established an impedance-based approach, which is able to differentiate cytostatic from cytotoxic drugs by recording time-kinetics of compound-effects on NIH 3T3 fibroblasts. In particular, based on a set of 40 compounds with known *in vivo* findings, they established an algorithm, which analyzes the shape of the impedance curves to differentiate mechanisms of toxicity [8]. Finally, another advantage of such technology is that compounds with similar mode of action (e.g., nuclear hormone modulators, anti-mitotic, DNA damaging, protein synthesis inhibitor compounds) can produce similar impedance-based time-dependent cell response profiles (TCRP) [9]. 

Impedance-based TCRP has been used to measure and characterize cellular responses to antimitotic compounds [7]. Ke *et al.* [7] screened a compound library and identified novel antimitotic compounds, with the majority confirmed by independent assays, based on clustering analysis of the TCRPs. In other applications, impedance measurement was successfully used to measure cytotoxic effects in alveolar type II cells and vascular endothelial cells [10], human astrocytic cells [11], neuronal cell lines [12] and human epithelial intestinal HT-29 cell line [13]. Our data indicate that the methodology is also extremely useful to determine the best coating and cellular density conditions for different adherent cellular models, including HepG2, ND7/23, mouse cardiomyocytes and fibroblasts [14]. In addition, reproducibility was also optimal when HepG2 cells were exposed to 0.1% dimethyl sulfoxide (DMSO) and to 0.0025% triton X-100 in 31 independent experiments, as well as when cardiomyocytes and fibroblasts were exposed to 21 compounds in three different experiments [14].

Despite the evident assets of the xCELLigence platform, many validation studies are still required to better evaluate this quite recent technology. For instance, it was shown recently that a cell index decrease is not always associated with cytotoxicity effects and that there are some confounding factors that can bring confusions in the analysis [14]. The objective of this study was to further assess the usefulness of the RTCA and, in particular, the xCELLigence platform. The objectives were to (i) compare cell index generated by RTCA and cell viability measured with a traditional cytotoxicity assay in primary human and rat hepatocytes, as well as in HepG2 and HepaRG cells exposed to 50 compounds, (ii) determine if compounds with similar mechanisms of action produce specific profiles in HepG2 and HepaRG cells exposed to 17 reference compounds and (iii) evaluate the predictivity of the genotoxicity signatures (specificity and sensitivity evaluation) determined by impedance with a set of 81 proprietary UCB compounds in HepG2 cells.

## 2. Materials and Methods

### 2.1. Chemicals Tested

All compounds were ordered from Sigma-Aldrich (Saint-Louis, MO, USA), except celecoxib and teniposide, which were purchased from Sequoia Research Products (Pangbourne, UK). Fresh concentrated stock solutions were prepared in dimethyl sulfoxide (DMSO) immediately before first use and then kept at −20 °C for potential retesting.

### 2.2. Quality Control: Test of Different Coating Conditions and Cell Titration Test

Different experiments were performed to determine the optimal culture conditions for each cellular model, except for the cryopreserved HepaRG. For this latter model, the provider of the cells recommended to work at very high cell density (*i.e.*, 100,000 cells/well). For the fresh HepaRG cells, Biopredic performed the cell seeding, according to their internal procedures. For the other cell types (*i.e.*, HepG2, primary Human and rat hepatocytes), the following coating conditions were used: no coating, collagen (Collagen R Solution 0.2%; Serva, Heidelberg, Germany), fibronectin (Sigma-Aldrich, Saint-Louis, MO, USA) and poly-D-lysine (Sigma-Aldrich). E-plates were coated, according to the manufacturer’s recommendations. For each coating condition, different cell densities (0.625, 1.25, 2.5, 5, 10, 20, 40 and 80 × 10^3^ cells per well) were tested in triplicate. The best combination (coating and cell density) was selected according to two important criteria. Firstly, as recommended by the manufacturer (Roche Diagnostics), the cell index should be minimum 0.5 and ideally 1 (or above) before starting any experiment (*i.e.*, before cells are exposed to compounds). Secondly, the cell number selected should be in the cell index/cell number linear range. For more details, please refer to Atienzar *et al*. [14]. According to both criteria, the optimal and used conditions (cell density, coating) for the different cellular models are summarized below:

- HepG2 cells: 10,000 cells/well, collagen
- HepaRG cells (cryopreserved): 100,000 cells/well (as recommended by the cell provider), collagen
- Primary human hepatocytes: 20,000 cells/well, collagen
- Primary rat hepatocytes: 15,000 cells/well, collagen

Results were recently published for four different models, including HepG2, ND7/23, as well as mouse cardiomyocytes and fibroblasts cells [14].

### 2.3. Cellular Models Used, Cell Culture and Manipulation of the Cells

HepG2 cells: the human hepatocellular carcinoma cell line (HepG2) was purchased from the European Collection of Cell Cultures (ECACC, Salisbury, UK). Cells were maintained in Dulbecco’s modified Eagle’s medium supplemented with 10% fetal bovine serum, 2 mmol/L L-glutamine and 1× nonessential amino acid solution at 37 °C in a humidified 5% CO_2_/95% air atmosphere. Cells were passaged twice a week using 0.5% trypsin-EDTA. Cells were used between passage 19 and 35. 

HepaRG cells: experiments with HepaRG cells (human hepatoma cell line) were performed on (1) 96-well plates ready to use or (2) with cryopreserved HepaRG. Two different formats were used, because both experiments on HepaRG cells were not performed at the same time and the Biopredic format evolved from fresh to cryopreserved cells. Consequently, it was not possible to compare both formats, but we recently successfully used both fresh and cryopreserved HepaRG for gene expression/cytochrome P450 activity evaluation and cytotoxicity investigations, respectively [15]. (1) Plates ready to use were purchased from Biopredic International (Rennes, France). E-plates (Roche Diagnostics, Vilvoorde, Belgium) were sent to Biopredic, who performed the cell seeding according to their internal procedures. On arrival, HepaRG cells were handled according to the manufacturer’s recommendations. In brief, the medium was discarded, and 100 µL of low DMSO medium was added for 24 h. The low DMSO medium was then replaced by 100 µL of high DMSO medium for at least 2 days. On the day of the experiment, the high DMSO medium was replaced with the ‘enriched’ medium (without DMSO, proprietary medium), and the compounds were added, with 0.5% as a final DMSO concentration in each well. The plates were incubated at 37 °C in a humidified 5% CO_2_/95% air atmosphere). Cryopreserved HepaRG cells were purchased from Invitrogen (Life Technologies, Merelbeke, Belgium). The cells were handled according to the manufacturer’s instructions. In brief, HepaRG cells were thawed at 37 °C, poured in 9 mL of pre-warmed (37 °C) HepaRG^TM^ Thaw, Plate and General Purpose Working medium (Life Technologies’ proprietary medium). The cells were centrifuged at 360 g for 2 min at room temperature (RT), resuspended in 5 mL HepaRG^TM^ Thaw, Plate and General Purpose Working medium and cell viability was determined. The cells were seeded in a collagen-coated E-plate at a density of 100,000 cells/well (as recommended by the cell provider) and placed on the plate reader in a humidified 5% CO_2_/95% air atmosphere at 37 °C. The medium was renewed every 2–3 days for 8 days. At day 9, 10 µL of compound was added to the cells.

Primary Human Hepatocytes (HH): the cells were purchased from CellzDirect (Cheshire, UK). Cells were thawed based upon CellzDirect’s standard method. In brief, hepatocytes were thawed at 37 °C, poured into pre-warmed (37 °C) CHRM™ thawing medium (CellzDirect’s proprietary medium) at a ratio of one vial/50 mL. The cells were centrifuged at 100 g for 10 min, resuspended in 2–3 mL cold (4 °C) CHPM™ plating medium and cell viability was determined. The cells were seeded in a collagen-coated E-plate at a density of 20,000 cells/well and allowed to attach in a humidified 5% CO_2_/95% air atmosphere at 37 °C for *ca*. 4–6 h, after which the medium was changed with Williams E medium supplemented with dexamethasone and cocktail B (cat n° CM4000, Life Technologies). Subsequently, 10 μL of compound was added to the wells. 

Primary Rat Hepatocytes (HH): the cells were purchased from Life technologies (Gent, Belgium). Cells were thawed according to Life Technologies’ standard protocol. In brief, hepatocytes were thawed at 37 °C, poured into pre-warmed (37 °C) thawing-plating medium (11% serum, Williams E medium containing dexamethasone, as well as cocktail A (cat n° CM3000, Life technologies)) at a ratio of one vial/50 mL. The cells were centrifuged at 80 g for 10 min, resuspended in 2–3 mL cold (4 °C) thawing-plating medium and cell viability was determined. The cells were seeded in a collagen-coated E-plate at a density of 15,000 cells/well and allowed to attach in a humidified 5% CO_2_/95% air atmosphere at 37 °C for ca. 4–6 h, after which the medium was changed with Williams E medium supplemented with dexamethasone and cocktail B (cat n° CM4000, Life Technologies). 

### 2.4. Impedance, Cell Index Measurements [9]

To quantify cell status based on the measured cell-electrode impedance, a parameter termed cell index (CI) is derived, according to the following equation:

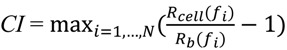

where *R_b_*(*f*) and *R_cell_*(*f*) are the frequency-dependent electrode resistances (a component of impedance) without cells or with cell present, respectively. *N* is the number of the frequency points at which the impedance is measured. Thus, cell index is a quantitative measure of the status of the cells in an electrode-containing well. Under the same physiological conditions, more cells attaching onto the electrodes lead to larger *R_cell_*(*f*) value and then to a larger value for cell index. Furthermore, for the same number of cells present in the well, a change in the cell status, such as morphology will lead to a change in the cell index. A “normalized cell index” at a given time point is calculated by dividing the cell index at the time point by the cell index at a reference time point. Thus, the normalized cell index is 1 at the reference time point. 

In our experiments, the normalization was always done by using the last timepoint before compound addition. This allows comparing more precisely the effect of the different concentrations tested *versus* the control. The cell index values presented in this manuscript were calculated from triplicate values (technical replicates) except for the cells exposed to DMSO (n = 6) and for the positive controls (n = 2, 0.00125, 0.0025 and 0.005% triton X-100). Data represent the average ± standard deviation. For more details concerning the RTCA measurement principle, please refer to Solly *et al.* [16]. For all experiments, the background reading was performed in presence of medium, except for the fresh HepaRG cells. For this latter model, the background was done in presence of the cells when the plates were received from Biopredic, who performed the cell seeding according to their internal procedures. Please see below for more details.

### 2.5. Generation of Different Signatures in HepG2 and (Fresh) HepaRG Cells

HepG2 plates were coated with collagen R. The background reading was performed in presence of 100 µL of medium. Then 90 µL of cells were added to obtain the adequate cell density (10,000 HepG2 cells per well). The E-plates containing the HepG2 cells were allowed to incubate at RT for 30 min and placed on the plate reader in the incubator for continuous recording of impedance overnight. After 16–24 h, the HepG2 cells were exposed to the compounds for at least 72 h. 

Experiments on fresh HepaRG cells were performed with HepaRG plates ready to use. The 96-HepaRG plates were handled as described in the previous “HepaRG cells” section. The background reading was done on the E-plates seeded with the cells. The background reading is normally done in absence of the cells, but since the E-plates were sent to Biopredic, to avoid any contamination problems, we decided not to perform the background reading in absence of cells. HepaRG cells were also exposed to compounds for at least 72 h.

Stock solutions of 100 mM (in DMSO) were used to prepare 3 other stock solutions at 10, 1 and 0.1 mM in 100% DMSO. These solutions were diluted 50 times in water. Then, 10 µL of these solutions were directly added into the wells containing 190 µL of medium. Final concentrations in the plates were 0.1, 1, 10 and 100 µM with 0.1% DMSO as final concentration. Compounds were added only once. Each condition was measured in triplicate except for the cells exposed to 0.1% DMSO (n = 6) and for the positive controls (n = 2, 0.00125, 0.0025 and 0.005% triton X-100). The cells were monitored in real-time, at 37 °C in a humidified 5% CO_2_/95% air atmosphere, using the multi-plate (96-well plate format) xCELLigence platform (Roche Diagnostics). Intervals for data collection were every 10 minutes after compounds addition for the first 2 h and every 30 min for at least 72 h.

Seventeen compounds were used in this experiment. Three to five compounds known to be calcium modulators, anti-mitotic agents, DNA damaging compounds and nuclear receptor agents were evaluated per category. The cellular models investigated were HepG2 and HepaRG cells. These latter were exposed for at least 72 h to 0.1, 1, 10 and 100 µM of compound. The objective was to determine if similar profiles were generated by compounds sharing the same mechanism of action and if the profiles were comparable to those generated in A549 (non-small lung cancer cells), as published in Abassi *et al.* [9]. A score of 1 or 0 was given when the profile was comparable or not, respectively, to those generated in the paper of Abassi *et al.* [9]. The main criteria used to compare the RTCA profiles was the shape of the curve, and this parameter was qualitatively evaluated by eye. Some of the RTCA signatures due to calcium modulators, antimitotics, DNA damaging agents and nuclear receptor modulators have been well characterized in A549 cells. In practice, a visual comparison was accomplished for HepG2 and HepaRG cells with the reference curves generated in A549 cells [9]. Indeed, the complete list of the clustered compounds shown in supplementary figures [9] helped to better define the typical RTCA profiles obtained in A549 cells for the different mechanisms of actions. 

### 2.6. Cytotoxicity: LC50 Calculation Comparison of Cell Index-Generated by RTCA and Cell Viability Measured by a Traditional Assay in HepG2, Cryopreserved HepaRG Cells, Primary Human and Rat Hepatocytes

Experiments on HepaRG were performed with cryopreserved HepaRG cells. Please refer to the “HepaRG cells” section for more details. HepG2 and cryopreserved HepaRG cells were seeded on coated collagen E-plates, according to the manufacturer’s recommendations. The background reading was performed in presence of 100 µL of medium. Then 90 µL of cells were added to obtain the adequate cell density per well (10,000 for HepG2 cells; 100,000 for HepaRG cells). For both cell types, the E-plates containing the cells were allowed to incubate at room temperature (RT) for 30 min and placed on the plate reader in the incubator for continuous recording of impedance overnight. After 16–24 h, the HepG2 cells were exposed to the compounds. HepaRG cells were cultured for 9–10 days before compound addition, according to CellzDirect’s recommendations. 

Primary rat and human hepatocytes were seeded on collagen R coated E-plates, according to the manufacturer’s recommendations. The background reading was performed in presence of 100 µL of medium. Then 90 µL of cells were added to obtain the adequate cell density per well (15,000 for rat hepatocytes; 20,000 for human cells). The E-plates containing the cells were allowed to incubate at RT for 30 min and placed on the plate reader in the incubator for continuous recording of impedance. After *ca*. 3 h attachment, the cells were exposed to the compounds.

The 4 cellular models were exposed to a set of 50 compounds for 5 consecutive days. The compounds were added twice at day 0 and day 3. Four concentrations using technical triplicates were used for each compound. The 50 compounds included 46 reference compounds (drugs known to be hepatotoxic in Humans or not), as well as 4 proprietary UCB compounds. The concentrations used were not fixed, but were selected based on Cmax values found in literature, as well as in commercial and proprietary UCB databases. Cmax refers to the maximal concentration of a compound in human blood. The concentrations tested were multiple of Cmax (*i.e*., 12.5 Cmax, 25 Cmax, 50 Cmax and 100 Cmax). 

Stock solutions (in DMSO) were prepared in the same manner as described in the section previously described “Generation of signature profiles in HepG2 and fresh HepaRG cells”. Each condition was measured in triplicate except for the cells exposed to DMSO (n = 6) and for the positive controls (n = 2, 0.00125, 0.0025 and 0.005% triton X-100). The cells were monitored in real-time, at 37 °C in a humidified 5% CO_2_/95% air atmosphere, using the multiplate xCELLigence platform. Intervals for data collection were every 10 min after compounds addition for the first 2 h and every 30 min for 5 days.

After 5 day exposure, impedance measurement was compared to cell viability using the Promega (Madison, USA) CellTiter-Fluor™ cell viability assay (reference G6080). For this purpose, the cells from the same E-plates were used to perform the cell viability assay. The CellTiter-Fluor™ Cell Viability Assay is a non-lytic fluorescence assay that measures the relative number of live cells. The live-cell protease activity is restricted to intact viable cells and is measured using a fluorogenic, cell-permeate, peptide substrate. In the plate, the culture medium was removed and replaced by fresh medium. The CellTiter Fluor reagent was added, according the manufacturer recommendations, and the fluorescence was measured. 

For the comparison, first of all, the averaged control values (*i.e.*, cells exposed to solvent) were set to 100% for both impedance and fluorescence parameters at the end of the experiments (*i.e*., after 5-day exposure). Then, the % of the cells exposed to the compounds at different concentrations was calculated relative to the control values. Finally, a coefficient of correlation was calculated for the impedance and fluorescence parameters using the Microsoft Excel 2010 software.

The % viability data from the impedance and fluorescence parameters were also used to calculate the LC_50_ (concentration that kills 50% of the cells) using a linear regression fit when applicable. In brief, the slope (a) and intercept (b) of the regression line was automatically calculated using the Microsoft Excel 2010 software with the data closest to the concentration killing 50% of the cells. The equation, y = ax + b, was then used to calculate the LC_50_. 

### 2.7. Evaluation of Impedance Output to Predict Genotoxicity Potential with Proprietary UCB Compounds in HepG2 Cells

Plates were coated with collagen R, according to the manufacturer’s recommendations. The background reading was performed in presence of 100 µL of medium. Then, 90 µL of cells were added to obtain the adequate cell density (*i.e*., 10,000 cells per well). The E-plates containing the cells were allowed to incubate at RT for 30 min and placed on the plate reader in the incubator for continuous recording of impedance overnight. After 16–24 h, the HepG2 cells were exposed to the compounds for 72 h.

Stock solutions were prepared at 200, 100, 50 and 25 mM (in DMSO) and further diluted to prepare other intermediate stock solutions (10% DMSO). Then, 10 µL of these solutions were directly added into the wells containing 190 µL of medium. Final concentrations in the plates were 125, 250, 500 and 1,000 µM with 0.5% DMSO as final concentration in the plate. Compounds were added only once. Each condition was measured in triplicate except for the cells exposed to 0.5% DMSO (n = 6) and for the positive controls (n = 2, 0.00125, 0.0025 and 0.005% triton X-100). The cells were monitored in real-time, at 37 °C in a humidified 5% CO_2_/95% air atmosphere, using the multiplate xCELLigence platform. Intervals for data collection were every 2 min after compounds addition for the first 2 h and every 30 min for 72 h. 

In this experiment, HepG2 cells were exposed for 72 h to 81 proprietary compounds belonging to two central nervous system projects in the field of neuropathic pain (35 and 46 compounds). The objective was to determine if the compounds that were identified as genotoxic (according to at least the Human GreenScreen assay [17] or possibly other assays, such as Chinese hamster ovary micronucleus assay [5] or the mini Ames test [18]) generated genotoxic profiles. A compound was classified as genotoxic according to the impedance measurements when, after compound addition, the cell index was higher compared to the control curve, followed by a decrease in cell index that reached at least 50% mortality within 48 h exposure. A compound was classified as negative when both conditions were not met. This rule was set based on cell index profiles that were obtained from reference compounds known to be genotoxic or not (preliminary experiments).

In order to evaluate the performance of the RTCA assay, the sensitivity, specificity and concordance (accuracy) were determined. The sensitivity is defined as the ability of a test system to predict the positive outcome under evaluation (*i.e*., genotoxicity). The specificity represents the ability of a test system to predict the negative outcome under evaluation (*i.e*., non-genotoxicity). The concordance or accuracy corresponds to the proportion of chemicals correctly identified by a particular test. 

## 3. Results

### 3.1. Example of RTCA Profiles Generated in HepG2 Cells Exposed to Six Compounds

**Figure 1:**
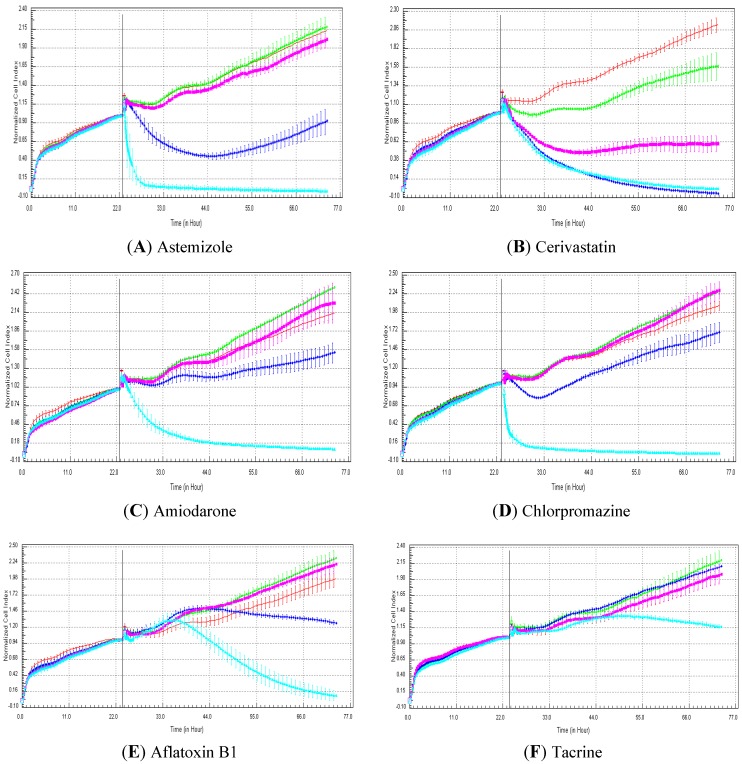
Examples of real-time cell analyzer (RTCA) profiles generated with hepatotoxic compounds in HepG2 cells. HepG2 cells were exposed to: 0 (0.5% dimethyl sulfoxide (DMSO), red curve), 0.1 (green curve), 1 (purple curve), 10 (dark blue curve) and 100 (light blue curve) µM with six different compounds. The drugs tested were astemizole (**A**), cerivastatin (**B**), amiodarone (**C**), chlorpromazine (**D**), aflatoxin B1 (**E**) and tacrine (**F**). Cell indexes were normalized with the last time point before compound addition. The normalized time point is indicated by the vertical line. Each data point was calculated from triplicate values (except for control cells n = 6). Data represent the average ± standard deviation. For more details, please refer to the Materials and Methods section.

Figure 1 shows examples of RTCA profiles generated in HepG2 cells exposed to astemizole (Figure 1(A)), cerivastatin (Figure 1(B)), amiodarone (Figure 1(C)), chlorpromazine (Figure 1(D)), aflatoxin B1 (Figure 1(E)) and tacrine (Figure 1(F)) at four concentrations (*i.e*., 0.1, 1, 10 and 100 µM). The rapid decrease in cell index, as shown for astemizole (Figure 1(A)) and chlorpromazine (Figure 1(D)) at 100 µM, is not associated to pure cytotoxicity effects. 

Indeed, such profiles are generally due to compounds modulating calcium and/or targeting cytoskeleton proteins. A typical cytotoxicity effect is represented, for instance, in Figure 1(B,C), in which HepG2 cells were exposed to cerivastatin and amiodarone, respectively. Indeed, the decrease in cell index displays a slope less pronounced than cell index decrease observed in HepG2 exposed to 100 µM of astemizole or chlorpromazine. Figure 1 shows examples of compounds with varying degrees of cytotoxicity. Out of the six compounds presented in Figure 1, the most cytotoxic one is cerivastatin (Figure 1(B)) (a severely hepatotoxic drug) with a concentration killing 50% of the cells (LC_50_) of 0.64 µM at 48 h. Tacrine, a moderately hepatotoxic drug, is considered as the least cytotoxic drug in HepG2 cells, with a LC_50_ close to the highest concentration tested (*i.e.*, 100 µM) (Figure 1(F)). Finally, aflatoxin B1 generated a genotoxic RTCA profile in HepG2 cells at 100 µM (Figure 1(E)). 

### 3.2. Examples of Cytotoxicity Data: RTCA Profiles Generated in HepG2, HepaRG, Primary Rat and Human Hepatocytes Exposed to Cerivastatin

**Figure 2:**
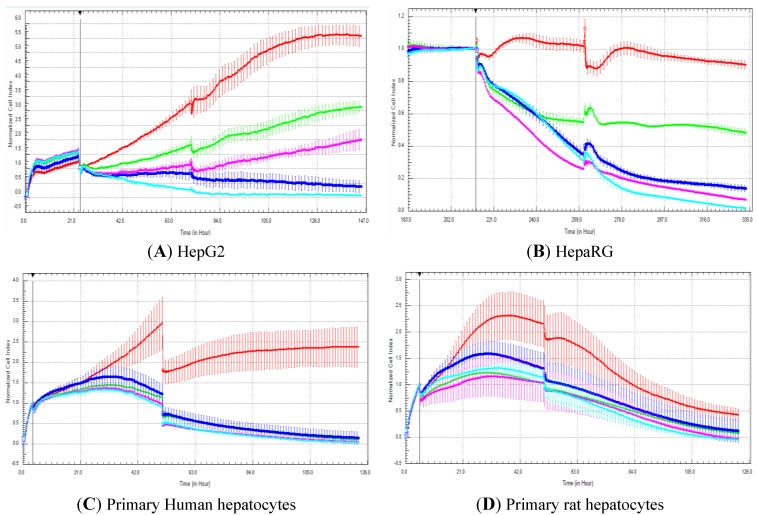
RTCA profiles generated in HepG2, cryopreserved HepaRG, primary rat and human hepatocytes exposed to cerivastatin. Cellular models as indicated in the panels **A**, **B**, **C** and **D**. Cells were exposed to: 0 (0.5% DMSO, red curve), 33 (green curve), 66 (purple curve), 132 (dark blue curve) and 264 (light blue curve) µM cerivastatin for five days. The concentration tested were multiple of the Cmax value (Cmax cerivastatin = 2.64 µM), *i.e*., 12.5, 25, 50 and 100 Cmax. Cerivastatin was added on day 0 (close to the vertical line) and day two. Cell indexes were normalized with the last time point before compound addition. The normalized time point is indicated by the vertical line. Each data point was calculated from triplicate values (except for control cells n = 6). Data represent the average ± standard deviation. For more details, please refer to the Materials and Methods section.

The RTCA curves show that the cell index of control cells exposed to 0.5% DMSO increased and/or stabilized for all cell types up to five days of treatment except for the primary rat hepatocytes (Figure 2). Indeed, for this latter model, the cell index started to decline particularly after two-days’ exposure (Figure 2(D)). Dose response relationships were obtained particularly for HepG2 cells (Figure 2(A)) and to a lesser degree for HepaRG cells (Figure 2(B)) exposed to cerivastatin. Significant cytotoxic effects were observed for all concentrations tested in primary human hepatocytes exposed to cerivastatin (Figure 2(C)). For all the cellular models tested, clear cytotoxic effects were already observed after the first exposure period at all cerivastatin concentrations tested. The addition of cerivastatin a second time on day two did not induce a more significant decrease in cell index (Figure 2) for the four cellular models. 

### 3.3. Cytotoxicity Data: Comparison of Impedance (RTCA) and Cell Viability (Traditional Assay) Readouts

One of our objectives was to evaluate whether cytotoxicity data generated from RTCA curves were comparable to cell viability data measured with the CellTiter-Fluor™ cell viability assay (Promega). Primary rat and human hepatocytes, as well as HepG2 and HepaRG cells, were exposed to 50 compounds, and both parameters (*i.e*., RTCA and cell viability) were compared after five-days’ exposure. Table 1 gives a summary of the correlation for all the cellular models tested. A satisfactory relationship was obtained for HepG2 and cryopreserved HepaRG cells, with a correlation of 88.5 and 73.3%, respectively. Nevertheless, a lower association was observed with primary human and rat hepatocytes, with a correlation of 51.8 and 57.6%, respectively.

### 3.4. Generation of Signature Profiles in HepG2 and HepaRG Cells

In this experiment, three to five compounds known to be either calcium modulators, antimitotics agents, DNA damaging compounds or nuclear receptor agents were evaluated for each category in HepG2 and fresh HepaRG cells (Table 2). 

**Table 1:** Correlation between impedance and cell viability in different cellular models exposed to a set of 50 compounds. The cellular models, as indicated in table, were exposed to four concentrations of compounds in triplicate. Averages and correlation (%) were calculated with Excel software. For more details, please refer to the Materials and Methods section.

Cellular models	Origin	Correlation (%)
HepG2	Human	88.5
Cryopreserved HepaRG	Human	73.3
Primary hepatocytes	Human	51.8
Primary hepatocytes	Rat	57.6

**Table 2:** Effect of different mechanisms of action on cell index curves (RTCA) generated in HepG2 and fresh HepaRG cells. Three to five compounds known to be either calcium modulators, antimitotics agents, DNA damaging compounds or nuclear receptor agents were evaluated per category. A score of 0 or 1 was given when the RTCA profiles generated were comparable or not to those generated in the paper of Abassi *et al.* [9]. A global score is given, as well as the % of success to reproduce the signatures published in Abassi *et al.* [9]. HepG2 and HepaRG cells were exposed to a set of 17 compounds for at least 72 h to 0 (0.5% DMSO), 0.1, 1, 10 and 100 µM. Cell indexes were normalized with the last time point before compound addition. Each data point was calculated from triplicate values. For more details, please refer to the Materials and Methods section.

Compounds tested	Signatures			
	Reproduced in		Types	Description
	HepG2	HepaRG		
Celecoxib	0	0	Calcium modulators	Rapid CI decrease
Tamoxifen	1	1		
Fluphenazine	1	1		
Methiothepin	1	1		
Perphenazine	1	1		
Colchicine	1	1	Antimitotic	Wavy and flat CI
Nocodazole	1	1		
Noscapine	0	1		
Teniposide	1	1	DNA damaging	Increase in CI compared to control followed by CI decrease (apoptosis)
Camptothecin	1	0		
Strophanthidin	0	1		
Etoposide	1	0		
Benzo(a)pyrene	0	1		
Budesonide	1	1	Nuclear receptor	CI higher than control CI
Hydralazine	1	1		
Hydrocortisone	1	1		
Methylprednisolone	1	1		
Total score/17	13	14		
% of success	76.5	82.4		
Total score/17	16			
HepG2 + HepaRG				
% of success	94.1			
HepG2 + HepaRG				

**Figure 3:**
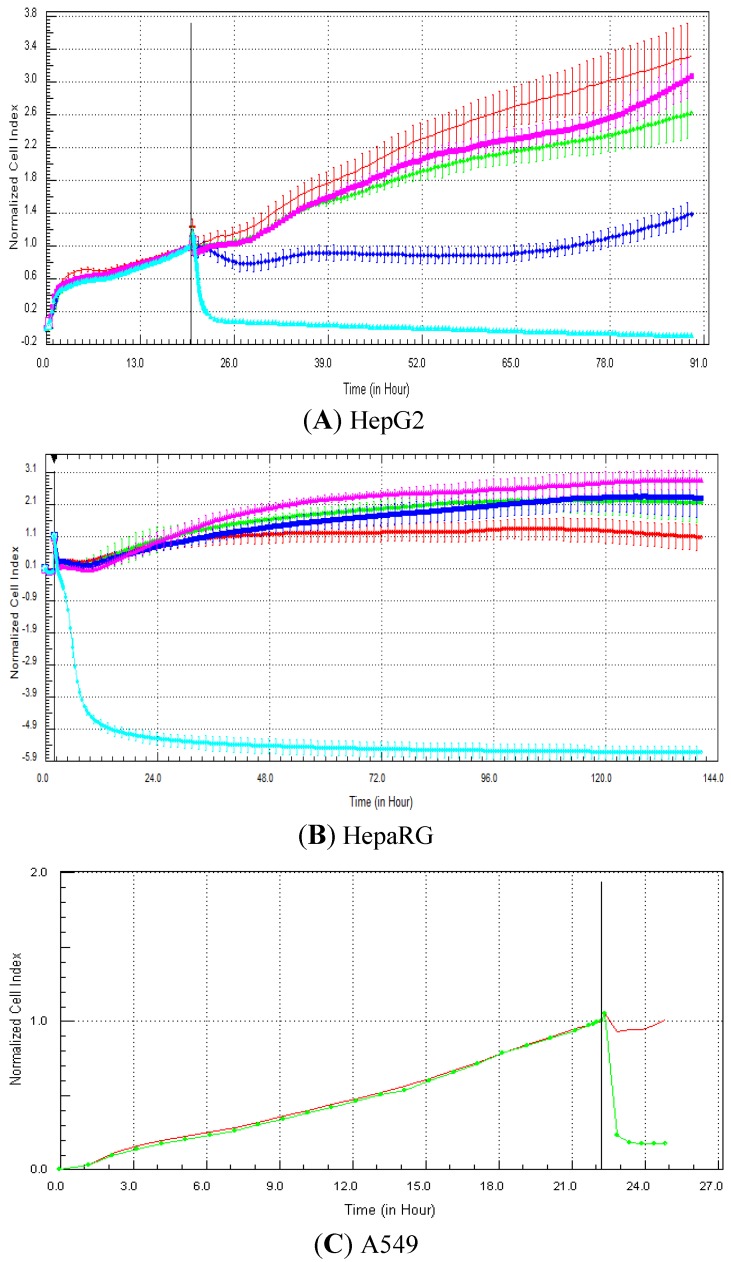
Effect of tamoxifen (calcium modulator) on cell index curves (RTCA) in HepG2, fresh HepaRG and A549 cells. HepG2 (**A**) and HepaRG (**B**) cells were exposed for at least 72 h to 0 (0.5% DMSO, red curve), 0.1 (green curve), 1 (purple curve), 10 (dark blue curve) and 100 (light blue curve) µM and A549 cells to 0 (control DMSO, red curve) and 20 µM (green curve) of tamoxifen (**C** from Abassi *et al.* [9]). Cell indexes were normalized with the last time point before compound addition. Panels A and B: each data point was calculated from triplicate values (except for control cells n = 6). Data represent the average ± standard deviation (except for panel C). The normalized time point is indicated by the vertical line. For more details, please refer to the Materials and Methods section.

**Figure 4:**
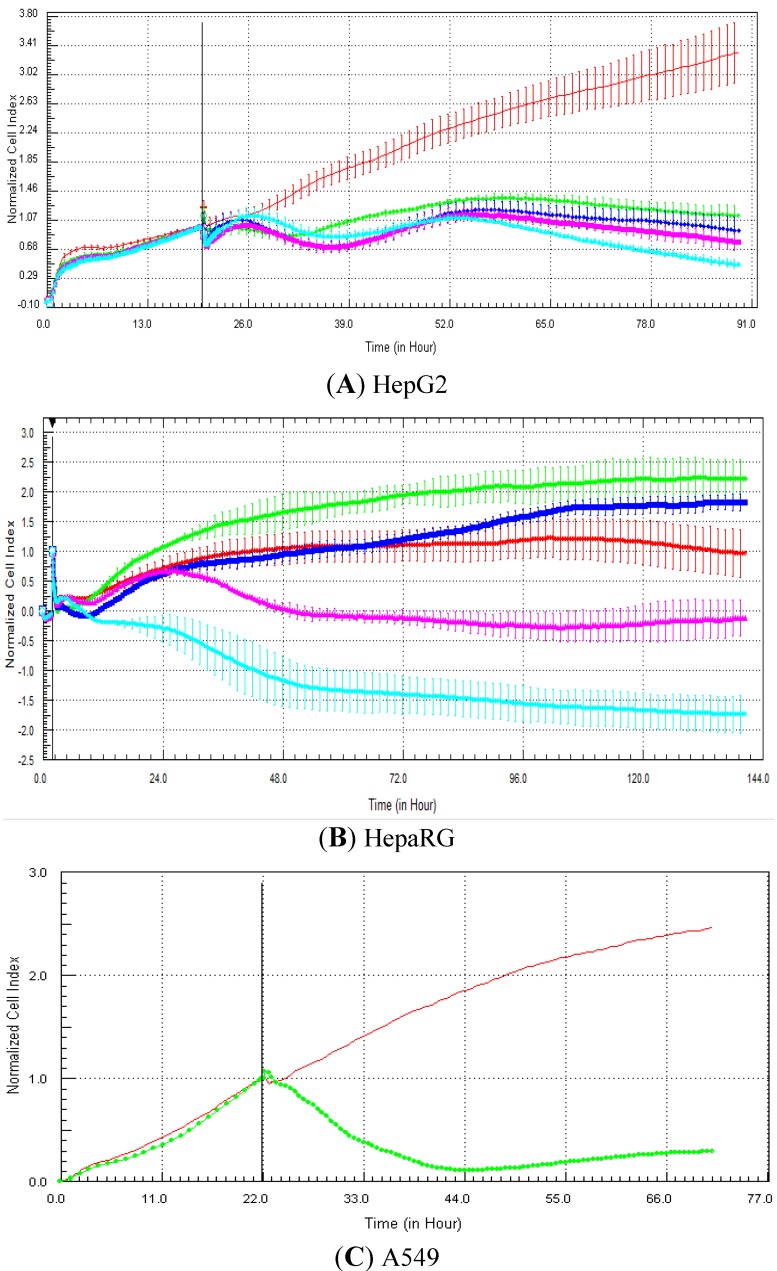
Effect of nocodazole (antimitotic) on cell index curves (RTCA) in HepG2, fresh HepaRG and A549 cells. HepG2 (**A**) and HepaRG (**B**) cells were exposed for at least 72 h to 0 (0.5% DMSO, red curve), 0.1 (green curve), 1 (dark blue curve), 10 (purple curve) and 100 (light blue curve) µM and A549 cells to 0 (control DMSO, red curve) and 20 µM (green curve) of nocodazole (**C** from Abassi *et al.* [9]). Cell indexes were normalized with the last time point before compound addition. Panels A and B: each data point was calculated from triplicate values (except for control cells n = 6). Data represent the average ± standard deviation (except for panel C). The normalized time point is indicated by the vertical line. For more details, please refer to the Materials and Methods section.

**Figure 5:**
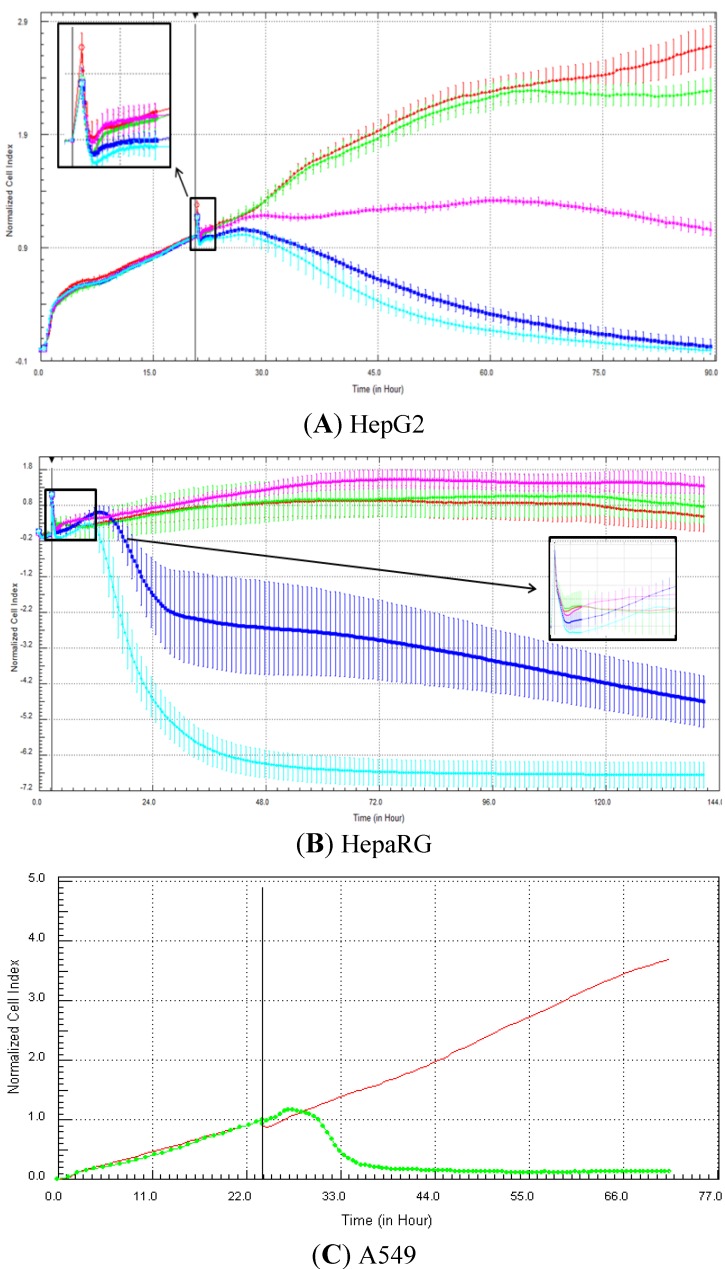
Effect of strophanthidin (DNA damaging) on cell index curves (RTCA) in HepG2, fresh HepaRG and A549 cells. HepG2 (**A**) and HepaRG (**B**) cells were exposed for at least 72 h to 0 (0.5% DMSO, red curve), 0.1 (green curve), 1 (purple curve), 10 (dark blue curve) and 100 (light blue curve) µM and A549 cells to 0 (control DMSO, red curve) and 20 µM (green curve) of nocodazole (**C** from Abassi *et al.* [9]). Cell indexes were normalized with the last time point before compound addition. Panels A and B: each data point was calculated from triplicate values (except for control cells n = 6). Data represent average ± standard deviation (except for panel C). The normalized time point is indicated by the vertical line. For more details, please refer to the Materials and Methods section. A zoom is provided in case of overlapping curves.

The objective was to determine if similar RTCA profiles could be generated with compounds sharing the same mechanism of action in these two cellular models. A score of 1 or 0 was given when the profile (produced in HepG2 and fresh HepaRG cells) was comparable or not, respectively, to those generated in A549 cells (non-small lung cancer cells) reported in the paper of Abassi *et al.* [9]. We have decided to use the profiles generated in A549 cells, because the publication [9] gives access to profiles generated with a large number of compounds (Figure 1, Figure 2, Figure 3, Figure 4, Figure 5). Please refer to the discussion for more details. Twenty µM was tested in the paper of Abassi *et al.* [9], whereas a range of concentrations (0.1, 1, 10 and 100 µM) was used in the present manuscript. Indeed, these concentrations in the range 0.1–100 µM are commonly tested to populate our internal databases. Overall, 76.5 and 82.4% of signatures were reproduced in HepG2 and HepaRG cells, respectively, compared to A549 cells. Interestingly, when the HepG2 and HepaRG results were combined, the % of success reached 94.1% (*i.e.*, only 1/17 compound was not detected in both models *i.e*., celecoxib). Similar results were obtained in both cellular models with calcium modulators and nuclear receptor compounds. All four compounds targeting nuclear receptors produced specific profiles in both cellular models (Table 2). Four out of five compounds known to be calcium modulators also generated typical RTCA profiles in both models. Nevertheless, different responses were also obtained in both models for 5/17 compounds tested. For instance, noscapine gave an antimitotic specific RTCA profile in HepaRG cells, but not in HepG2 cells, whereas camptothecin generated a DNA damaging profile in HepG2 cells, but not in HepaRG cells (Table 2).

**Figure 6:**
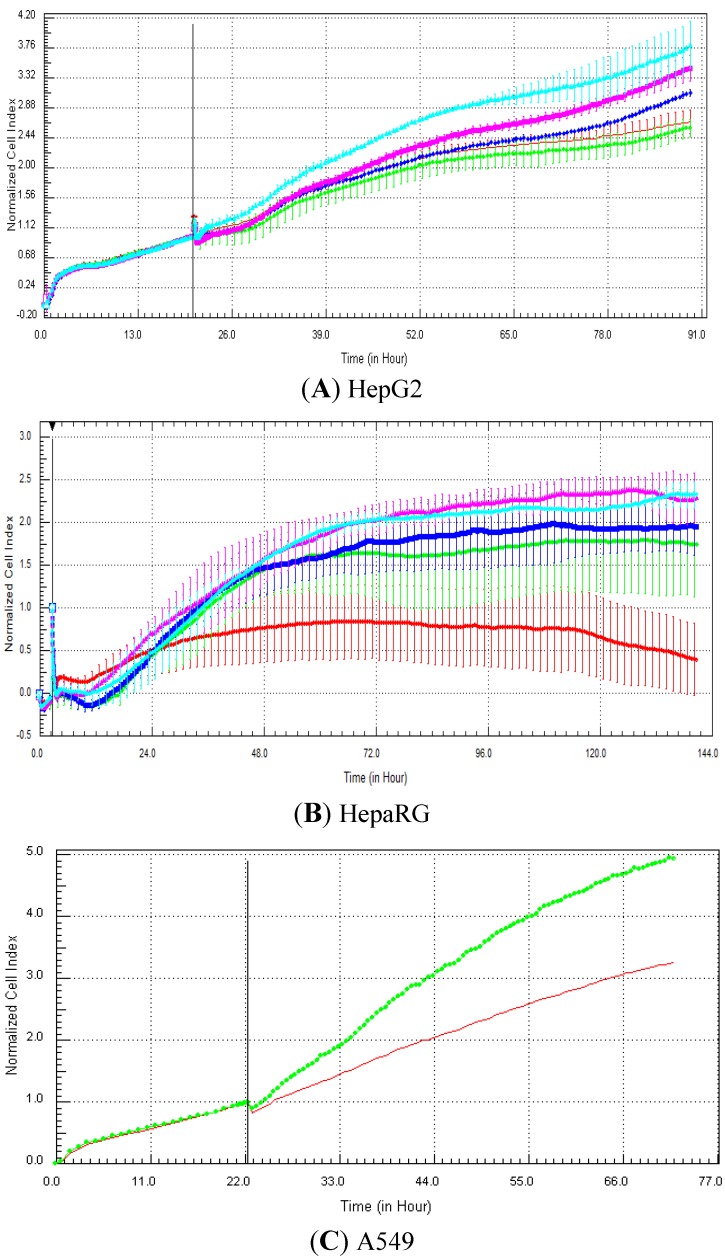
Effect of methylprednisolone (nuclear receptor modulator) on cell index curves (RTCA) in HepG2, fresh HepaRG and A549 cells. HepG2 (**A**) and HepaRG (**B**) cells were exposed for at least 72 h to 0 (0.5% DMSO, red curve), 0.1 (green curve), 1 (dark blue curve), 10 (purple curve) and 100 (light blue curve) µM and A549 cells to 0 (control DMSO, red curve) and 20 µM (green curve) of methylprednisolone (**C** from Abassi *et al*., [9]). Cell indexes were normalized with the last time point before compound addition. Panels A and B: each data point was calculated from triplicate values (except for control cells n = 6). Data represent the average ± standard deviation (except for panel C). The normalized time point is indicated by the vertical line. For more details, please refer to the Materials and Methods section.

The specific RTCA patterns obtained in HepG2, fresh HepaRG and A549 cells exposed to one of the compounds belonging to each of the four mechanisms of action (*i.e*., tamoxifen, nocodazole, strophanthidin and methylprednisolone) are presented in Figure 3, Figure 4, Figure 5, Figure 6, respectively. It is noteworthy that for the fresh HepaRG cells, the background reading was done on the E-plates seeded with the cells. The background reading is normally done in absence of the cells, but since the E-plates were sent to Biopredic, to avoid any contamination problems, we decided not to perform the background reading in absence of cells. Consequently, as the reference index value is zero (in presence of the cells), negative values can be obtained in case of cytotoxic effects or specific signatures, such as calcium modulators. For the HepG2 cells, the lowest cell index value was zero (in case of 100% cell mortality), as the plates were prepared in house and the background measurement was performed with 100 µL medium in absence of cells. Typically, compounds that control calcium efflux induce a decrease in cell index immediately after compound addition. This effect was observed in A549 cells exposed to 20 µM tamoxifen (Figure 3(C)), as well as in HepG2 (Figure 3(A)) and HepaRG (Figure 3(B)) cells at 100 µM. It takes generally a few hours (e.g., 5–10 h) for the cell index to reach its minimum value, which can last for a few days (Figure 3). Antimitotic compounds produce a flat and/or wavy cell index kinetic, due to the inhibition of the cell division. In other words, cells stay alive, but do not proliferate. This signature was detected in HepG2 cells exposed to all concentrations of nocodazole (0.1–100 µM; Figure 4(A)), in HepaRG cells at 10 and 100 µM (Figure 4(B)) and in A549 cells at 20 µM (Figure 4(C)). Compounds that damage DNA can produce an increase in cell index (more or less pronounced) compared to control, followed by a decrease in cell index. This specific pattern was observed in HepaRG cells exposed to 10 µM strophanthidin (Figure 5(B)), in A549 cells at 20 µM (Figure 5(C)), but not in HepG2 cells in the range 0.1–100 µM (Figure 5(A)). Finally, the compounds that target nuclear receptors induce generally an increase in cell index in comparison to control that can last for a few days. This signature was observed in HepG2 cells exposed to 1–100 µM methylprednisolone (Figure 6(A)), in HepaRG cells at all concentrations tested (0.1–100 µM) (Figure 6(B)) and in A549 cells at 20 µM (Figure 6(C)). 

### 3.5. Evaluation of Impedance Output to Predict Genotoxicity Potential with Proprietary Compounds

In this experiment, HepG2 cells were exposed for 72 h to 81 proprietary UCB compounds belonging to two central nervous system projects in the field of neuropathic pain (35 and 46 compounds). The objective was to determine if the compounds that were identified as genotoxic according to a battery of *in vitro* genotoxicity assays generated genotoxic RTCA profiles. The predictivity data (sensitivity, specificity and concordance) are presented in Table 3. Interestingly, in both central nervous system (CNS) projects the specificity (*i.e.*, the detection of the negative compounds) was high (minimum 92.3%). Nevertheless, the detection of the genotoxic compounds was totally different in both projects. In the first CNS project, the sensitivity reached 63.6% (*i.e.*, 14/22), whereas none of the seven genotoxic compounds were identified as genotoxic based on the RTCA profile in the second CNS project. When both projects were taken into account, the sensitivity, specificity and concordance were 48.3% (14/29), 96.2% (50/52) and 79% (64/81), respectively. 

**Table 3:** Evaluation of RTCA predictivity to detect genotoxicity with a set of 81 proprietary UCB compounds. HepG2 cells were exposed for at least 72 h to 81 proprietary UCB compounds belonging to two central nervous system projects (CNS1 and CNS2). The concentrations tested were 125, 250, 500 and 1,000 µM (unless solubility problems were encountered). The objective was to determine if the compounds that were identified as genotoxic by traditional genotoxicity *in vitro* assays generated RTCA genotoxic profiles. A compound was classified as genotoxic according to the impedance measurements when after the compound addition, the cell index generated by the compound was higher than the control curve followed by a decrease in cell index that reached at least 50% mortality within 48 h exposure. A compound was classified as negative when both conditions were not met. Examples of typical genotoxicity profiles are presented in Figure 7(A,B) (*i.e.*, HepG2 and A549 cells exposed to etoposide, respectively). For more details about the definition of the terms (sensitivity, specificity and concordance/accuracy), please refer to the Materials and Methods section.

	CNS project 1	CNS project 2	CNS project 1 & 2
Number of compounds tested	35	46	81
Genotoxic compounds	22	7	29
Non-genotoxic compounds	13	39	52
Genotoxic signature (RTCA)	14	0	14
Non-genotoxic signature (RTCA)	12	38	50
Sensitivity	63.6% (14/22)	0% (0/7)	48.3% (14/29)
Specificity	92.3% (12/13)	97.4% (38/39)	96.2% (50/52)
Concordance (accuracy)	74.3% (14 + 12/35)	82.6% (0 + 38/46)	79.0% (14 + 50/81)

**Figure 7:**
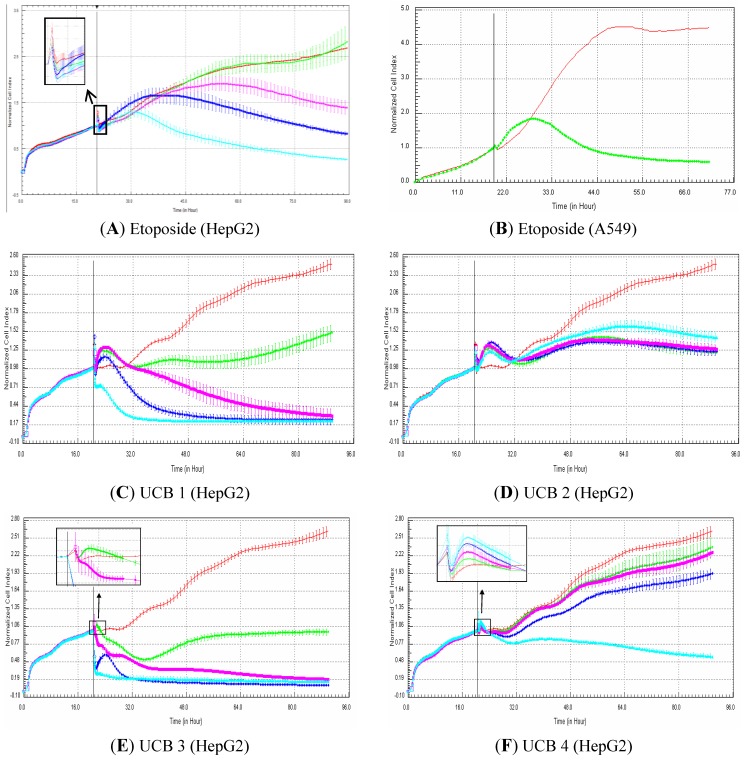
Example of RTCA profiles generated with etoposide (A549 and HepG2 cells) and proprietary UCB compounds (HepG2 cells). (**A**) HepG2 cells were exposed to: 0 (0.5% DMSO, red curve), 0.1 (green curve), 1 (purple curve), 10 (dark blue curve) and 100 (light blue curve) µM of etoposide. (**B**) A549 cells exposed to 0 (control DMSO, red curve) and 20 µM (green curve) of etoposide (Abassi *et al*. [9]). (**C**–**F**): HepG2 cells were exposed to 0 (0.5% DMSO, red curve), 125 (green curve), 250 (purple curve), 500 (dark blue curve) and 1,000 (light blue curve) µM of compound UCB 1, 2, 3 and 4, respectively. Cell indexes were normalized with the last time point before compound addition. The normalized time point is indicated by the vertical line. All panels (except B): each data point was calculated from triplicate values (except for control cells n = 6). Data represent the average ± standard deviation (except for panel B). A zoom is provided in case of overlapping curves. For more details, please refer to the Materials and Methods section.

Examples of RTCA graphs generated with etoposide and proprietary UCB compounds are presented in Figure 7. The effect of etoposide (a reference genotoxic compound) on cell index is presented in Figure 7(A,B) in HepG2 and A549 cells [9], respectively. According to our classification criteria (see Materials and Methods section for more details), etoposide was classified as positive in HepG2 cells (at 10 and 100 µM) and in A549 cells (at 20 µM). The effect of four UCB compounds is also presented in Figure 7. Compound 1 was identified as genotoxic at 125, 250 and 500 µM (Figure 7(C)), whereas compounds 3 and 4 were classified as genotoxic at 125 (Figure 7(E)) and 1,000 µM (Figure 7(F)), respectively. Finally, despite the typical genotoxic profile, compound 2 was considered to be non-genotoxic, as none of the concentrations tested induced 50% mortality in comparison to the control value (Figure 7(D)). Nevertheless, this compound could be considered as equivocal, as the 50% mortality was nearly reached for all concentrations tested (Figure 7(D)).

## 4. Discussion

The use of label-free technologies [19] (based on acoustic resonance, electrical impedance, microcantilevers, nanowires or differential calorimetry) applied to cell biology and drug discovery is receiving more and more attention. The present manuscript focuses on impedance measurement, which is used to gather information on diverse cellular processes, including proliferation, migration, cytotoxicity and receptor-mediated signaling [20]. Our recent data indicated that the xCELLigence platform (based on impedance) was useful to determine the best coating and cellular density conditions for four different adherent cellular models, including hepatocytes, cardiomyocytes, fibroblasts and hybrid neuroblastoma/neuronal cells [14]. In addition, cell index data were highly reproduced when cardiomyocytes and fibroblasts were exposed to 21 compounds in three independent experiments [14]. Finally, we have also demonstrated that cytotoxicity data determined by RTCA were comparable to cell imaging markers (*i.e.*, number of cells) when HepG2 and HepaRG cells were exposed to 21 drugs [14]. Nevertheless, despite the widely use of label-free technologies, there is a lack of publications looking at the correlation between cellular impedance measurements and classical toxicity endpoints, such as ATP, cell number and viability markers. Consequently, one of our objectives was to compare cell viability generated with the xCELLigence platform with the CellTiter-Fluor™ Promega assay in four different cellular models exposed to 50 compounds. In addition, our intention was also to evaluate if compounds with similar mechanisms of action produced similar profiles with a set of 17 compounds in HepG2 and HepaRG cells and, finally, to assess the predictivity of the RTCA genotoxicity signatures with a set of 81 UCB proprietary compounds in HepG2 cells.

In the drug discovery paradigm, there is a shift from biochemical-based screening to cell-based phenotypic screening [21]. This is mainly due to the fact that cell-based phenotypic assays more closely recapitulate the complexity of whole organism and can bring information on cellular toxicity. Cells have the advantage to be fully functional entities with membrane and intra-cellular connections, while they are relatively easy to use. The integration of phenotypic profiling technologies, combined with effective experimental and *in silico* target identification approaches, can improve success rates of lead selection and optimization in drug discovery [21]. In addition, the combination of phenotypic screening and multi-omics-based target identification and validation approaches will provide an effective approach to discover new bioactive small molecules, their target protein and mechanism of actions [22]. In this context, the cell imaging platform, as well as label-free technologies based on impedance measurement, represent powerful tools for cell-based phenotypic screening. In a recent study, the RTCA signature obtained in neuroblastoma (ND7/23) cells exposed to cytochalasin B (a modulator of tubulin polymerization) was compared to cell-imaging data to better understand the mechanisms of action at the cellular level [14]. Clearly, the combination of both technologies may represent a powerful approach to gain information on mechanisms of action [14].

In the present manuscript, primary rat and human hepatocytes, as well as HepG2 and HepaRG cells, were exposed to 50 compounds to compare cell index generated by RTCA and cell viability measured with a standard, commercially available assay after five days of compound exposure. The commercial test refers to the CellTiter-Fluor™ cell viability assay, which is commonly used by the scientific community [23]. In our experiments, cellular models were exposed twice (two administrations of compounds over five days in total) to compounds to determine if this would allow detecting more hepatotoxic compounds in comparison to single exposure. Nevertheless, in general, the double exposure approach did not pick up more hepatotoxic compounds (Figure 2 and data not shown). A correlation of 88.5 and 73.3% was obtained with HepG2 and HepaRG cells, respectively. This is well in agreement with our previous study, where a high correlation (*i.e*., 87%) was obtained between cell index generated by RTCA and the cell number determined by cell imaging in HepG2 and HepaRG cells exposed to 21 compounds after three days of exposure (one administration of compounds over three days) [14]. Nevertheless, lower association was observed in primary human and rat hepatocytes with a correlation of 51.8 and 57.6%, respectively. With the rat primary hepatocytes, a decrease in cell index was observed after two-days’ treatment, even in control cells. Consequently, the predictivity of the rat primary hepatocytes should only be evaluated after two-day and not after five-day exposure. Nevertheless, this fact does not explain the low correlation after five-day exposure between impedance and the CellTiter-Fluor™ assay. In our hands, lower LC_50_ values were obtained in primary human and rat hepatocytes according to impedance values compared to cell viability measured with the fluorescent assay (data not shown). Interestingly, a study stated that impedance measurement is a convenient and reliable method for the detection of proliferation and kinetics of cell death in neuronal cell lines (e.g., HT-22 cells: immortalized hippocampal neurons) [12]. Nevertheless, the same study reported that impedance measurement is less suitable for the assessment of neuronal differentiation and viability of primary neurons (e.g., primary cortical cells) [12]. The main reason for this apparently lies within the different cellular integrity and adhesion characteristics, which favors a rounding up and detachment of intact cellular bodies in HT-22 cells, contrasting to the cellular disintegration and leftovers of primary cells remaining after cell death [12]. Another study reported that the proliferation of normal human epidermal keratinocytes (NHEK) measured by impedance-based method did not correlate with other more traditional approaches [24]. This could be accounted to the specific morphological appearance of these cells [24]. Consequently, such data clearly emphasize the need to perform quality control experiments before launching any further investigations with different cell types. Indeed, strict quality control of cells is required for the standardization and interpretation of results in all areas of cell-based research, especially in drug discovery [25]. 

One of our objectives was to determine if specific profiles were generated in HepG2 and HepaRG cells exposed to reference compounds having different mechanisms of actions (calcium modulators, nuclear receptor, antimitotic and DNA damaging agents). The analysis of the signatures was performed by visual inspection, due to the lack of an interpretation/classification algorithm. Nevertheless, we consider that the approach is not optimal, as the classification and interpretation of profiles depend on the end-user experience, and, thus, are subjected to human approximation (particularly for difficult profiles). Clearly, the development of a reliable analysis tool is needed to standardize the interpretation of RTCA patterns [14]. Out of the 17 compounds tested, 76.5 (13/17) and 82.4 (14/17)% of specific profiles were reproduced in HepG2 and HepaRG cells, respectively. Interestingly, the combination of results from both cellular models (*i.e*., HepG2 and HepaRG cells) allowed to generate specific profiles (in at least one cellular model) for 94.1% of the compounds tested (*i.e*., 16/17). This illustrates the need to use different cellular models to better identify potential mechanisms of action, because intracellular targets are not necessarily represented and/or expressed in all types of cells. In addition, since we used 10-fold dilution series (0.1, 1, 10 and 100 µM), it could be argued that the use of a narrower range of concentrations could allow generating a higher percentage of signatures with both cell lines. 

Calcium modulators are known to induce a brutal decrease in cell index, whereas cytotoxic compounds produce a decrease in cell index, which is less severe. As a consequence, the analysis of the slope may allow to easily differentiate between cytotoxic and calcium modulator agents. Nevertheless, the effect of weak calcium modulators on impedance may be challenging to predict, due to potentially confounding cytotoxicity effects. Recently, 2,000 compounds (50% Food and Drug Administration approved, 29% natural products, 18% bioactive compounds, 3% herbicides and insecticides) were screened on the xCELLigence platform on A549 non-small lung cancer cells and PC3 prostate cancer cell lines [9]. For instance, 160 and 190 compounds led to early changes in profiles compared to control in PC3 and A549 cells, respectively, with 75 common hits between both cell types [9]. Abassi *et al.* indicated that some signatures, such as the nuclear receptor-like responses, appear to be cell type-specific, as different profiles were obtained with both cell types [9]. Thus, by using different cell types for screening and profiling, it may be possible to cover a sufficiently large subset of intracellular targets to gain valuable information on target and off target effects. In another study, Ke *et al.* screened *ca*. 120,000 compounds from a library to generate impedance-based time-dependent cell response profiling (TCRP) [7]. This approach allowed to identify novel antimitotic compounds with 113/117 hit compounds confirmed as antimitotic, based on independent assays [7]. 

In the present study, the sensitivity and specificity of the xCELLigence platform to detect genotoxic compounds were evaluated on 81 UCB proprietary compounds (29 genotoxic compounds and 52 non-genotoxic compounds) from two CNS projects, in the field of neuropathic pain, in HepG2 cells. Since single exposure is usually performed with genotoxicity screening assays, such as the GADD45a-GFP GreenScreen Human Cells assay (GHC) [26] and Ames microplate format (AMPF) [27], HepG2 cells were exposed to compounds using single administration. Specificity was high in both projects, with a minimum of 92.3% (12/13) in the first project. This suggests that the rate of false positive is low and this is particularly important in the context of early screening in drug discovery. With regard to the detection of the positive compounds, the sensitivity was 63.6% (14/22) in the first CNS project, whereas none of the seven genotoxic compounds were identified as such in the second one. When compounds from both projects were considered together, the sensitivity, specificity and concordance were 48.3% (14/29), 96.2% (50/52) and 79% (64/81), respectively. Despite the modest sensitivity, this is quite interesting for the screening of compounds, considering that the xCELLigence platform can give information on cytotoxicity, as well as on potential on target and off target effects. Furthermore, it is noteworthy that we tested UCB proprietary compounds in HepG2 cells in absence of the S9 fraction (which contains diverse metabolism enzymes), whereas the GHC [26] and AMPF [27] tests are performed in presence and absence of S9. Consequently, sensitivity could have been improved by using S9 fraction with the HepG2 cells and/or by testing the compounds in HepaRG cells, which are metabolically competent [15]. In addition, more data are needed to better evaluate the potential of the xCELLigence platform to efficiently detect genotoxic compounds particularly in the drug discovery paradigm. According to the signatures, genotoxic compounds induce, in comparison to control, an increase in cell index for approximately 12 h that is followed by a rapid decrease in signal. The hypothesis is that, in case of DNA damage, all damaged cells are arrested at the DNA damage checkpoint before they enter into the S-phase. In other words, all the cells are synchronized at cell cycle stage, where cells are on average bigger than control cells (which are a mixture of cells of different sizes at G1, G2, S,…, phases). This would explain why cell index generated from the damaged cells is higher than cell index in control cells. In case the damaged cells cannot efficiently repair their DNA, apoptosis is induced, and thus, the cells die, which correlates with a decrease in cell index. Consequently, the use of dividing cells (e.g., HepG2) is recommended for the detection of genotoxic signatures. Finally, the use of primary cells, which do not divide, is not recommended to detect genotoxic compounds based on RTCA profiles. 

In conclusion, since cell-based phenotypic assays are starting to be more used in drug discovery, label-free technologies based on impedance measurement represent a powerful tool. The present study reveals a good correlation (*ca*. 80%) between cell index generated by RTCA and cell viability measured by a standard and traditional assay in HepG2 and HepaRG cells exposed to a set of 50 compounds. Nevertheless, low correlation (*ca*. 55%) was observed in primary human and rat hepatocytes exposed to the same set of compounds. Specific profiles were generated in HepG2 and HepaRG exposed to most of the reference compounds with different mechanisms of action (calcium modulators, nuclear receptor modulators, antimitotic and DNA damaging agents). Finally, the predictivity of the xCELLigence platform was investigated for the detection of genotoxic and non-genotoxic compounds with a set of 81 proprietary UCB compounds. The data indicates a sensitivity of *ca*. 50% and a high specificity (close to 100%), indicating that the label-free technologies based on impedance can be used in drug discovery for a first genotoxicity screen. Overall, despite some limitations, the RTCA technology is a powerful and reliable tool in drug discovery for toxicity and pharmacology studies.
